# Effects of Predrying and Spontaneous Fermentation Treatments on Nib Acidification, Fermentation Quality, and Flavour Attributes of Ghanaian Cocoa (*Theobroma cacao*) Beans

**DOI:** 10.1155/2024/5198607

**Published:** 2024-06-10

**Authors:** Lukeman Haruna, Ernest E. Abano, Ernest Teye, Isaac Tukwarlba, Wilson Yeboah, Kesse J. Agyei, Mary Lukeman

**Affiliations:** ^1^ Department of Agricultural Engineering University of Cape Coast, Cape Coast, Ghana; ^2^ Quality Control Company (QCC) Limited Western North Regional Office, Sefwi Wiawso, Ghana; ^3^ Department of Plantation Cocoa Research Institute of Ghana (CRIG), New Tafo, Ghana; ^4^ Food and Drugs Audit Food and Drugs Authority (FDA) of Ghana, Accra, Ghana

## Abstract

Cocoa bean acidification, fermentation, and flavour quality are intricately shaped by pulp preconditioning and fermentation treatments. This study investigates the impact of predrying and subsequent fermentation on key parameters such as pH, titratable acidity, fermentation quality (% purity), fermentation index (FI), and overall flavour quality (global quality (GQ)) of cocoa beans. Extended predrying periods and fermentation durations demonstrated a significant enhancement in bean acidification, reflected in the rise of nib pH (6.61–7.33) and the decline in nib acidity (0.023–0.013 meg NaOH/100 g). Notably, the cut test underscored the substantial improvement in % purity, reaching 75.6–99.7% for beans predried at 2–8 hours followed by a 6-day of fermentation. FI increased significantly from 1.026 to a peak of 1.067, followed by a decline to 0.098 in the control, 6 hours, and 8 hours of predried beans, respectively. Sensory evaluation showed substantial improvement in the GQ (40.1–44.6) of beans predried at 2–8 hours and fermented for 6 days, compared to the control (38.3). In addition, a significantly higher preference was shown for cocoa liquor made from the beans predried for 4-6 hours and fermented for 6 days. Principal component analysis clustered samples according to the predrying time, fermentation duration, and quality parameters measured. Optimal conditions for enhanced nib acidification, fermentation quality, and flavour attributes were identified at 6-hour predrying and 6-day fermentation using the response surface methodology. The study highlights the potential of predrying as a pulp preconditioning technique for enhancing fermentative and final bean quality.

## 1. Introduction

Cocoa (*Theobroma cacao* L.) stands as a pivotal agricultural commodity, acclaimed for its commercial value, nutritional richness, and health benefits [1–4]. Despite their esteemed status, fresh cocoa beans exhibit extreme inherent bitterness and astringency, attributable to their relatively elevated polyphenol and methylxanthine content [5, 6]. They also lack the coveted brown chocolate colour and flavour associated with their fermented counterparts when roasted [7]. Fermentation enhances the potential development of these attributes by forming colour and flavour precursor compounds in the beans [8]. The presence and abundance of these compounds indicate the effectiveness of fermentation and influence the final flavour quality of the beans [9].

Cocoa seeds, enclosed within undamaged pods, are surrounded by a mucilaginous pulp abundant in fermentable sugars and citric acid [10]. As fermentation unfolds, yeasts, lactic acid bacteria (LAB), and acetic acid bacteria (AAB) successively act on the pulp, leading to liquefaction and the breakdown of its sugars into a blend of migratory metabolites (ethanol, lactic acid, and acetic acid) [11–13]. The AAB-produced acetic acid triggers a substantial temperature increase due to its exothermic nature [14, 15]. This, combined with the diffusion of organic acids—particularly acetic acid—into the beans, leads to seed death [16–18]. Simultaneously, the release of endogenous enzymes, following disruptions in the cell membrane due to the seed's death, initiates the degradation of storage carbohydrates and proteins [19, 20]. This enzymic activity yields flavour precursors, including reducing sugars and free amino acids, which are prerequisites for flavour compound formation in Maillard and Strecker degradation reactions, yielding chocolate note-producing aldehydes and pyrazines [21, 22]. Additionally, inherent polyphenol compounds undergo enzymic oxidation, transforming into quinones and tannins—essential for the formation of the characteristic chocolate brown colour [3, 8, 23]. Ultimately, the reduction in nib polyphenol content contributes to improvements in flavour quality, marked by diminished bitterness and astringency [24].

Previous studies on cocoa bean fermentation and flavour development underscore the significance of residual nib acidity in fermentation effectiveness and the final quality of beans [25–27]. Nib acidification directly influences microbial involvement, affecting the type and quantity of migratory metabolites generated during the fermentation process [28]. Furthermore, high nib acidity affects the activities of endogenous enzymes during precursor compound formation, which may eventually mask the flavour of the final product [9]. Hence, elevated nib acidity during fermentation, linked to excessive initial pulp levels, necessitates pulp preconditioning of freshly harvested cocoa beans for improved fermentative effectiveness and flavour quality [29, 30].

While conventional methods like pod storage exist, they prove laborious, time-consuming, and prone to risks of spoilage, pilfering, and seed contamination [31–35]. Furthermore, the associated prolonged storage period (3-7 days) may lead to an increased generation of defective beans during fermentation, compromising the final quality and flavour of the beans after drying [19]. Although results from less common techniques such as beans spreading on a preheated surface [36], depulping by centrifugation [10], enzymatic actions [36], and mechanical depulping [37] were promising, they present challenges in terms of cost and technical complexity [10]. In contrast, predrying emerges as a viable alternative, involving the sun exposure of freshly harvested beans for a brief period. This technique harnesses natural sunlight, minimizing costs, environmental impact, and risks associated with other methods [36]. By focusing on predrying as a pulp preconditioning technique, this study is aimed at enhancing fermentation effectiveness and flavour quality in Ghanaian cocoa beans.

## 2. Materials and Methods

### 2.1. Materials

Cocoa fermentation experiments were conducted in October 2022 at the Cocoa Research Institute of Ghana (CRIG), located in New Tafo, Eastern Region, Ghana. Cocoa pods used for the experiments were of the mixed hybrid variety.

#### 2.1.1. Harvesting, Pod Breaking, and Predrying

Ripe and healthy mature cocoa pods were manually harvested from the experimental plot, specifically K8 (latitude: 6.231906; longitude: -0.350051) and G15 (latitude: 6.230603; longitude: -0.349467) at CRIG. Shortly after harvesting, the pods were split open. Fresh cocoa beans, along with adhering pulp, were extracted using nonsanitized hands and then thinly spread on fresh perforated plantain leaves. The beans underwent sun drying under ambient conditions (28.1 ± 5.9°C and RH: 81.8 ± 17.0%) for 0, 2, 4, 6, and 8 hours. To ensure consistent pulp dehydration, the beans were turned every hour throughout the predrying process.

#### 2.1.2. Fermentation Treatments and Sampling

Approximately 60 kg of predried beans were heaped on perforated plantain leaves spread out in a circular shape on a fermentation platform. The fermentations occurred spontaneously and lasted for 0, 2, 4, and 6 days, each carried out in independent triplicates. Manual turning of beans was performed every 48 hours from the initiation of fermentation until its completion. Samples were randomly selected from the center of the fermenting heap on days 0, 2, 4, and 6. The samples were sun-dried until the moisture content reached ≤7.5%. Subsequently, the dried beans were sealed in black airtight plastic bags and kept in an odour-free dark room at 23–25°C until needed for further analyses.

#### 2.1.3. Experimental Design

The study employed a 5 × 4 general full factorial design, with predrying time (0, 2, 4, 6, and 8 hours) and fermentation duration (0, 2, 4, and 6 days) being the principal factors. The key parameters examined included the acidification (pH and titratable acidity), cut-test results, fermentation index (FI), and FQ scores of the bean samples. These analyses aimed to evaluate the final acidity, fermentative characteristics, and flavour attributes of the beans.

### 2.2. Methods

#### 2.2.1. Determination of Nib Acidification

The pH and titratable acidity (TA) of beans were determined following the procedures outlined by [38], with slight modifications. Approximately 10 g of dried nibs were homogenized in 90 ml of hot boiling ultrapure water, stirred manually for 30 seconds, and filtered with Whatman No. 4 filter paper. The resulting filtrate was then cooled to 23–25°C, and the pH of 25 ml aliquots was determined using a benchtop digital pH meter (Model S213 Mettler Toledo, Mettler Company Limited, Geneva, Switzerland). Calibration was performed with standard buffer solutions (pH = 3.50, 7.00, and 9.50). For TA, a titration of 10 ml aliquot with 0.1 N NaOH solution to an endpoint of 8.1 was conducted, and the results were expressed as the meg of the titrant per 100 g of the sample.

#### 2.2.2. Fermentation Quality (% Purity) and Fermentation Index (FI) during Cocoa Bean Fermentation

*(1) Determination of % Purity*. The cut test served as an index to evaluate fermentation effectiveness (level of brown bean occurrence) and bean wholesomeness (extent of defectiveness), following the method stated by [39] with slight modifications. Approximately 300 cocoa beans were longitudinally sliced into two halves using a small pocket (Okapi) knife to expose the internal nib surface. Each half underwent an examination for colour and quality defects. A slaty surface denoted a lack of fermentation (equation (1)). A purple surface (equation (2)) indicated incomplete fermentation, while a light to fully brown surface indicated a well-fermented bean (equation (3)). A mouldy, germinated, or weevily cut surface was considered unwholesome and, following the protocols of the ISO 2451:2017 [40] standard, handpicked along with ineffectively fermented (slaty and purple) beans as defective. Subsequently, the picked beans were grouped, counted, and quantified separately based on identified defects as shown in equations (1)–(8). The quantification of purity (%) was determined using equation (9).

(1) $$\begin{array}{c} \%\text{Slaty}=\frac{\text{number of slaty beans picked}}{\text{total number of beans cut}}\times 100, \end{array}$$

where %slaty is the percentage occurrence of slaty beans in the samples.

(2) $$\begin{array}{c} \%\text{Purple}=\frac{\text{number of purple beans picked}}{\text{total number of beans cut}}\times 100, \end{array}$$

where %purple is the percentage occurrence of purple beans in the samples.

(3) $$\begin{array}{c} \%\text{Brown}=\frac{\text{number of brown beans picked}}{\text{total number of beans cut}}\times 100, \end{array}$$

where %brown is the percentage occurrence of brown beans in the samples, signifying fermentative effectiveness.

(4) $$\begin{array}{c} \%\text{Germinated}=\frac{\text{number of germinated beans picked}}{\text{total number of beans cut}}\times 100, \end{array}$$

where %germinated is the percentage occurrence of germinated beans in the samples.

(5) $$\begin{array}{c} \%\text{Mouldy}=\frac{\text{number of mouldy beans picked}}{\text{total number of beans cut}}\times 100 \end{array}$$

where %mouldy is the percentage occurrence of mouldy beans in the samples.

(6) $$\begin{array}{c} \%\text{Weevily}=\frac{\text{number of weevily beans picked}}{\text{total number of beans cut}}\times 100, \end{array}$$

where %weevily is the percentage occurrence of weevil-infested beans in the samples.

(7) $$\begin{array}{c} \text{All other defect}=\frac{\text{total germinated}+\text{total weevily}}{\text{total number of beans cut}}\times 100, \end{array}$$

(8) $$\begin{array}{c} \text{Wholesomeness of beans}=100-\left(\text{total mouldy}+\text{all other defects}\right), \end{array}$$

(9) $$\begin{array}{c} \%\text{Purity}=100-\left(\text{total mouldy}+\text{total purple}+\text{total slaty}+\text{all other defects}\right), \end{array}$$

where purity (%) is the fermentation quality of bean samples.

*(2) Determination FI*. Cocoa beans' fermentation effectiveness was spectroscopically confirmed by assessing the FI of the beans according to the method described by [41], with little modifications. Colour pigments were extracted from 0.5 g of ground nib using a 5 ml CH_3_OH:HCl (97 : 3 *v*/*v*) solution. The homogenate underwent cooling at 4°C for 16 hours on an orbital shaker and was centrifuged at 3500 × g for 5 minutes. The absorbance of the supernatant was measured at 460 nm and 530 nm wavelengths for anthocyanidin and anthocyanin content, respectively, using a UV/Vis spectrophotometer (Beckman Coulter DU 730 Life Science, MA, USA). The FI was calculated based on the ratio of absorbance at 460 nm to that at 530 nm.

#### 2.2.3. Sensory Evaluation of Cocoa Liquor

*(1) Sample Preparation and Cocoa Liquor Formation*. Sensory analysis was performed as described by [23], with some modifications. The dried samples were sealed in airtight transparent plastic bags and stored in an odour-free, dark room at 25–28°C (RH: 6.5–7.8%) for 2 months to allow for nib acidity stabilization and settling of intrinsic volatile compounds. Approximately 300 g of beans were roasted in a Stabil-Therma Gravity Oven (Model OV-18A, Blue M Electric, IL, USA) for 23 minutes, using an air temperature of 118°C. After cooling, 100 g of beans were fragmented and winnowed to separate nibs from shells. The nibs were milled into cocoa liquor using a Stepan mixer and then sieved. Subsequently, 2 g of cocoa liquor was filled into cubic plastic cups and maintained at 18°C for 5 days to allow for aroma stabilization before sensory evaluation.

*(2) Sensory Analysis*. Sensory analysis of the cocoa liquor involved a panel of 7 well-trained evaluators (comprising 4 females and 3 males, with an average age of 27 and 31 years, respectively) using a factorial statistical design that incorporated hidden reference liquors. All panelists had prior experience in evaluating the sensory profiles of cocoa-based products and provided free prior informed consent. Samples underwent quality control to ensure safety and wholesomeness. Next, labeled cubed samples were melted at 35°C; rested for an hour; presented to panelists in a quiet, spacious, well-lit (artificial lightening), and odour-free (activated carbon-filtered) temperature-controlled room (21 ± 1°C and RH: 50 ± 5%); and isolated from external influences (auditory, visual, and olfactory distractions) to compare with internal reference standards for flavour descriptors. Approximately 1 g of each sample was presented to each panelist in a clean 1 m^2^ off-white coloured sensory booth with shadow-free illumination equivalent to office lighting intensity levels of 700 lux to taste and evaluate sensory attributes based on the following flavour descriptors: cocoa flavour, fruity note, floral note, acidity note, bitterness, astringency note, and off-flavour note. To minimize perception fatigue, a maximum of 4 test samples were evaluated in each session to arrive at the sensory profile. Warm water was provided for rinsing the mouth in between tasting samples. Following the tasting, panelists indicated the flavour quality scoring of each sample based on a quantitative descriptive analysis (QDA) on a 0–10 scale (0: extremely bad; 10: excellent) to assess whether samples were better, equal, or inferior to the reference standards. The preference score (flavour quality) for each descriptor, scored by each panelist, was averaged and pooled for the overall flavour quality (global quality) ratings of each sample.

#### 2.2.4. Statistical Analysis

Data analysis was conducted using Minitab statistical software version 19.1 (MSS, LLC, USA). An analysis of variance (ANOVA) was performed to compare means, and the least significant difference (LSD) was employed to separate means, with significance accepted at the 5% confidence level (*p* < 0.05). Additionally, response surface methodology was employed to analyze the combined effects of predrying time (PT) and fermentation duration (FD) on the studied quality parameters. Models were developed using stepwise multiple regression procedures to establish relationships between PT and FD and the assessed parameters. Reported were the coefficients of the variables in the models and their contribution to the model's variation. Model adequacy was assessed using *R*^2^ values, where an *R*^2^ of at least 60% was considered indicative of a good fit. Furthermore, principal component analysis (PCA) was conducted to visually represent the complex data matrix and study the relationship between process parameters and response variables. The numerical optimization approach was employed to determine the ideal PT and FD, along with the composite desirability index, using Minitab statistical software version 19.1 (MSS, LLC, USA). All treatments and analyses were performed in triplicates, and results are reported as mean ± standard deviation.

## 3. Results and Discussion

### 3.1. Effect of Predrying on Nib Acidification during Cocoa Bean Fermentation

#### 3.1.1. Changes in pH

**Figure 1:**
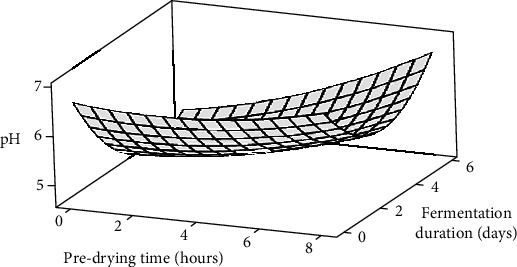
Surface plot showing the effects of predrying time and fermentation duration on the pH of cocoa beans.

The impact of predrying on nib pH during fermentation is depicted in Figure 1. Overall, a decline in nib pH was observed during fermentation across all PT levels. In line with previous studies [42, 43], a significant (*p* < 0.05) decrease from the initial pH of 6.61 to a minimum (4.64) at day 4, followed by a slight rise to a final value of 5.28 in the last 2 days of fermentation, was noted (Table 1). Consistent trends in nib pH variations were evident at all PT levels during fermentation, with final nib pH levels increasing significantly (*p* < 0.05) from 5.28 to 7.33 following 6-day fermentation in the 0-hour (untreated) and 8-hour PT treatment samples, respectively. The modest increase in bean pH during the latter stage of fermentation (day 4 to day 6) is crucial for determining fermentative and final flavour quality [30]. The suggested optimal range for colour and flavour precursor generation is pH 5.0–5.5 [6]. Enzymes like carboxypeptidases and aspartate-endoproteases work optimally in this pH range, selectively degrading the 7S Vicilin Class Globulin proteins during fermentation [2]. In this study, predrying significantly (*p* < 0.05) influenced pH increases in the later stages of fermentation, aligning them within the recommended range for the 4 and 6-hour PT treatments. This implies that predrying cocoa beans for 4–6 hours and fermenting for 6 days may enhance nib acidification for optimal fermentative and final flavour quality.

ANOVA of the data revealed that the observed variations in pH were significantly (*p* < 0.05) influenced by increasing PT and FD, along with their interactions (Table 2). Regression analysis demonstrated that both linear and quadratic terms of PT and FD, along with their interactions, significantly (*p* < 0.05) impacted the pH of the beans (Table 3). This indicates a curvilinear (full quadratic) relationship between these factors and the pH values at all sampling levels. The developed model could account for over 87% of the variations in bean pH (Table 3), implying that 13% of the variations were attributable to extraneous factors beyond the scope of this study.

#### 3.1.2. Titratable Acidity

The pH trend showed an inverse correlation with the TA levels, as outlined in Table 1. Significantly lower TA levels (*p* < 0.05) were observed in samples predried at various durations compared to the untreated sample. Notably, among the treated samples, TA values decreased significantly (*p* < 0.05), ranging from 0.062 to 0.013 meg NaOH/100 g for the 2–8-hour PT treatments after 6 days of fermentation. Predrying exposed the freshly harvested beans to ambient environmental conditions and microbes for an extended period (2-8 hours), potentially leading to increased production of pulp sweatings and loss of pulp moisture, resulting in reduced pulp volume per seed. This extended exposure may enhance the aeration and oxygen penetration of the bean-pulp mass during fermentation [44, 45]. Consequently, it may promote the respiration of available pulp sugars by microbes in the early fermentation stages rather than their direct fermentation into ethanol [31]. This initial microbial respiration could contribute to lower ethanol production, a prerequisite for acetic acid formation during the later stages of fermentation, resulting in reduced acidity levels in the beans [46–48]. This explains the significantly (*p* < 0.05) lower TA values (0.033–0.013 meg NaOH/100 g) observed in the 4–8-hour PT samples fermented for 6 days.

**Figure 2:**
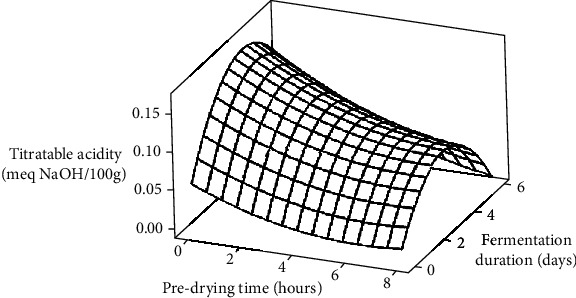
Surface plot showing the effects of predrying time and fermentation duration on the titratable acidity (meg NaOH/100 g) of cocoa beans.

The results, depicted in Figure 2, demonstrated that fermentation significantly (*p* < 0.05) increased TA levels from an initial 0.023 meg NaOH/100 g to a peak of 0.193 meg NaOH/100 g within the first 4 days, followed by a sharp decrease to 0.083 meg NaOH/100 g by day 6. Similar TA variation trends were observed across all predrying treatments. These findings align with the observations of Afoakwa et al. [46] and Amanquah [38], who reported similar TA variations during fermentation using pod storage and mechanical depulping, respectively, as pulp preconditioning techniques. The observed changes were attributed to the influx and subsequent decay of acetic acid from the cotyledon of the beans during fermentation [44, 47].

Statistical analysis (ANOVA) of the data revealed significant differences (*p* < 0.05) in acidity levels among fermenting beans with respect to PT, FD, and their interactions (Table 2). Regression analysis further demonstrated that the linear and quadratic effects of PT and FD had a significant (*p* < 0.05) influence on the TA levels of beans (Table 3). Additionally, significant (*p* < 0.05) interaction effects occurred between PT and FD. The calculated *R*^2^ value of 0.8353 indicated that the model accounted for 83.5% of the variability in acidity levels of the fermenting beans.

### 3.2. Effect of Predrying on Fermentation Quality (% Purity) and Fermentation Index (FI) of Cocoa Beans during Fermentation

#### 3.2.1. Changes in % Purity of Cocoa Beans

Generally, the % brown bean occurrence and wholesomeness of bean samples (absence of the occurrence of mouldy, germinated, and weevily beans) increased with FD at all levels of PT (Table 4). % Brown beans significantly increased (*p* < 0.05) from 0.0% to 50.7%; conversely, % slaty and % germinated beans (reflecting unwholesomeness) decreased from 87.7% to 0.7% and from 12.3% to 8.3%, respectively, for untreated beans after 6 days of fermentation. Comparable results were noted by [30, 48], albeit their studies utilized pod storage as a pulp preconditioning technique. The gradual transition in the cotyledon colour of unfermented beans from light purple to brown stemmed from the oxidation and polymerization of polyphenols during fermentation [9]. As elucidated by [42], anthocyanins—the polyphenol compounds responsible for the light purple colour of raw beans—are rapidly hydrolyzed by galactosidase to anthocyanidins and sugar molecules like arabinose and galactose. With improved oxygen build-up in the heap during fermentation, anthocyanidins undergo further oxidation by polyphenol oxidases to form quinones [49]. Subsequently, these reactive quinones engage in polymerization and condensation reactions with nitrogenous compounds such as proteins, short-chain polypeptides, and amino acids, resulting in the formation of insoluble, high-molecular-weight brown-pigmented tannins [50]. This process imparts the characteristic chocolate brown colour to the cotyledons, signifying the effectiveness of fermentation [7].

Moreover, the results revealed that increasing the PT led to an enhanced occurrence of brown beans. This occurrence increased significantly (*p* < 0.05) from 50.7% in the untreated beans to a maximum of 97.7% for the 6 hours of PT treatment after 6 days of fermentation. The trend in % brown bean occurrence was somewhat inversely mirrored by the trend in % purple bean occurrence. The occurrence of purple beans was significantly (*p* < 0.05) high (40.3%) in the untreated beans but reduced to a minimum of 0.3% in the 6-hour predried samples, followed by an increase to 12.7% in the 8-hour PT treatments. Purple bean formation occurs when fermentation terminates at the quinone stage of the polyphenol oxidation process [9]. Purple beans are therefore not considered defective beans since their quinone content may degrade further to tannin in the presence of sufficient oxygen during storage [51], hence indicating an incomplete fermentation process [52, 53].

The results (Table 4) also revealed a significant increase (*p* < 0.05) in bean wholesomeness with an increase in FD across all levels of predrying. Germinated beans decreased from an initial 12.3% to 8.3% at fermentation completion in the untreated bean samples. Predrying further improved the bean wholesomeness by reducing the occurrence of germinated beans to 5.0%, 2.0%, 0%, and 3.7% for the samples predried for 2, 4, 6, and 8 hours, respectively. Germination of cocoa beans occurs when the combined effects of diffused migratory metabolites, oxygen, and temperature during fermentation are insufficient to kill the seed embryo and arrest the development of emerging seed radicles [54]. The predrying treatments may have mitigated germinated bean occurrence during fermentation, potentially by improving microaeration in fermenting mass due to the initial low pulp volumes of the predried beans [36]. Germinated beans are deemed defective because the emerging radicle creates openings in the shells, facilitating the entry of mould spores and insects, whose activities may elevate free fatty acid levels in cocoa beans [40, 55]. The occurrence of mouldiness was notably observed in beans predried for 8 hours and fermented for 4 days or more. This occurrence could be attributed to the relatively higher microbial loads inoculated on pulps during predrying, along with the elevated levels of germinated beans produced by this treatment.

**Figure 3:**
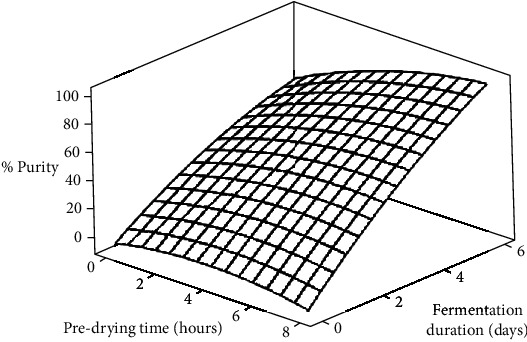
Surface plot showing the effects of predrying time and fermentation duration on % purity of cocoa beans.

The surface plot (Figure 3) illustrates the % purity of the beans during fermentation across all levels of predrying treatments. The % purity increased from 0% at the beginning of fermentation to 50.7%, 75.6%, 98%, 99.7%, and 82.7% for the untreated beans and 2 hours, 4 hours, 6 hours, and 8 hours for the PT-treated beans after 6 days of fermentation. As noted in [30], optimal cocoa flavour development typically occurs when % purity exceeds 60%, indicating that all levels of predrying produce adequately fermented cocoa beans. Moreover, this also suggests that the 4 to 6-hour predrying treatments yielded the most effectively fermented and wholesome cocoa beans after 6 days of fermentation. The general increase in % purity may be attributed to the progressive enhancements in fermentation effectiveness and wholesomeness of beans across all levels of predrying treatments throughout the fermentation process.

The analysis of variance (ANOVA) conducted on the data revealed significant (*p* < 0.05) differences in the levels of slaty beans, purple beans, brown beans, germinated beans, mouldy beans, and % purity scores of fermenting beans regarding PT, FD, and their interactions (Table 5). Regression analysis further demonstrated that both the linear and quadratic effects of PT and FD significantly (*p* < 0.05) influenced the % purity of the beans (Table 3). Additionally, significant (*p* < 0.05) interaction effects were observed between PT and FD. The high *R*^2^ of 0.919 indicates that the model could account for over 91% of the changes in % purity of the beans.

The analysis of variance (ANOVA) conducted on the data revealed significant (*p* < 0.05) differences in the levels of slaty beans, purple beans, brown beans, germinated beans, mouldy beans, and % purity scores of fermenting beans regarding PT, FD, and their interactions (Table 5). Regression analysis further demonstrated that both the linear and quadratic effects of PT and FD significantly (*p* < 0.05) influenced the % purity of the beans (Table 3). Additionally, significant (*p* < 0.05) interaction effects were observed between PT and FD. The high *R*^2^ of 0.919 indicates that the model could account for over 91% of the changes in % purity of the beans.

#### 3.2.2. Changes in FI of Cocoa Beans

**Figure 4:**
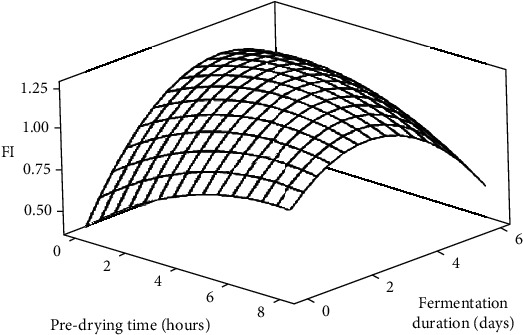
Surface plot showing the effects of predrying time and fermentation duration on the FI of cocoa beans.

FI, serving as a quality indicator, offers a more objective approach to gauging the extent of brownness in cocoa nibs and thus assessing the degree of bean fermentation [33]. It evaluates the level of anthocyanin degradation leading to anthocyanidin formation and subsequent polymerization into intricate tannins within cocoa nibs during fermentation [28]. Consequently, it quantifies the colour transformation of cotyledon from light purple to brown, providing insight into the degree of fermentation [32]. According to [56], well-fermented beans exhibit an FI ≥ 1, while lower values signify underfermented beans. Similar to the cut-test results, FI consistently increased with FD across all PT treatments (Figure 4). Initially, untreated beans displayed high absorbance at 530 nm, likely due to their elevated anthocyanin content, resulting in a low FI value of 0.669 at the onset of fermentation. However, during fermentation, the FI surged to a peak of 1.086 on day 4 before marginally declining to a final value of 1.026 after 6 days (Figure 4). This upswing in FI can be attributed to a notable rise in absorption at 460 nm, primarily stemming from increased condensation products of anthocyanins by day 4 of fermentation [28]. The subsequent dip in FI to 1.026 on day 6 may be attributed to the diminished solubility of condensation products of anthocyanins (e.g., cyanidin-3-galactoside and cyanidin-3-arabinoside), resulting in a slight reduction in absorption at 430 nm. Biehl et al. [36] similarly noted this trend, albeit employing pod storage as the pulp preconditioning technique in their study.

The results (Figure 4) also indicate that predrying led to slight increases in FI across all levels of FD, except for the 8-hour PT treatment, which recorded a lower FI value (0.908) after 6 days of fermentation. Additionally, the FI range of 1.02–1.07 for the 0–6 hour PT treatments suggests that the samples were fully fermented by day 6 of fermentation. These findings are consistent with the results of the cut test and suggest that beans predried for 4 to 6 hours were well-fermented on day 6 of fermentation, with the latter being the most effective. Conversely, the 8-hour treatment exhibited slight underfermentation, as confirmed by the cut-test results and the pH value of the beans after 6 days of fermentation.

Regression analysis of the data revealed a significant (*p* < 0.05) influence of the linear factor of FD, as well as the quadratic factor of PT and FD, on the FI of the beans (Table 3). Furthermore, significant (*p* < 0.05) interaction effects were observed between PT and FD on the FI of the beans. The model developed could explain over 61% of the variations in the FI of the beans, indicating that about 39% of the variations were attributable to other factors beyond the scope of this study (Table 3).

### 3.3. Effect of Predrying on Sensorial Attributes of Cocoa Beans during Fermentation

#### 3.3.1. Changes in Overall Flavour (Global) Quality (GQ) of Cocoa Beans

Overall flavour quality (GQ) serves as the most representative indicator of cocoa beans, encompassing various quality parameters during fermentation [23]. GQ is quantified as a composite score (*Q*_*P*_ + (10 − *Q*_*N*_) comprising the quality score for desirable (positive) flavour attributes (*Q*_*P*_) and the inverse quality score for negative flavour attributes (*Q*_*N*_) of cocoa beans.  Table 6 presents the changes in flavour attributes observed during fermentation across all predrying treatments. Fermentation significantly (*p* < 0.05) enhanced the GQ of cocoa beans at all predrying levels. Untreated beans exhibited the lowest score for positive attributes and the highest inverse quality score for negative flavour attributes at all FDs. Specifically, positive flavour attributes such as cocoa, fruity, and floral notes significantly (*p* < 0.05) increased from 2.0 to 6.3, 1.0 to 1.9, and 1.0 to 1.7, respectively, after 6 days of fermentation. Conversely, the inverse quality score for negative attributes significantly (*p* < 0.05) decreased from 9.0 to 6.9 for acidic flavour while increasing from 2.6 to 6.3 and 2.3 to 6.9 for bitterness and astringency notes, respectively, with stable scores for off-flavour sensations. Cocoa flavours, developed during roasting, stem from Maillard reactions between flavour precursor compounds like reducing sugars and free amino acids inherently generated during fermentation [57, 58]. The other desirable fruity and floral notes are also generated from these precursor compounds through various flavour-associated pathways during fermentation [59–61]. Consequently, a significant (*p* < 0.05) enhancement in the GQ score from 26.9 to 38.3 after 6 days of fermentation was observed (Table 6). Similar trends in flavour quality changes were noted across all predrying treatments. These progressive improvements in GQ could be attributed to the accumulation of flavour precursor compounds and reductions in polyphenolic compounds in cocoa beans during fermentation [42].

**Figure 5:**
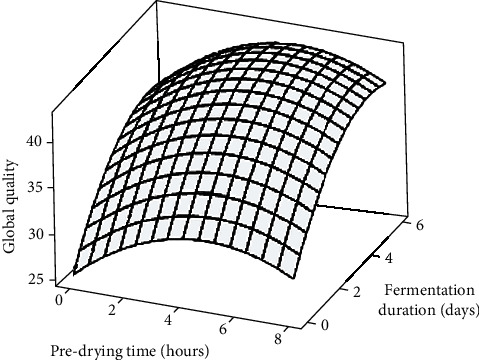
Surface plot showing the effects of predrying time and fermentation duration on the global quality of cocoa beans.

The surface plots generated (Figure 5) revealed a consistent increase in GQ with increasing PT across all FDs. The significant (*p* < 0.05) rise from an initial value of 26.9 to a GQ range of 38.3-44.6 for the 2–6-hour PT treatments confirmed that all samples, except for the 8-hour treatment (34.7), were fully fermented. However, the optimal flavour quality was attained with the 6-hour PT and 6-day fermentation treatments. Although sensory evaluation results showed no significant impact of predrying on the global quality score of the 8-hour PT treatment, it was intriguing to observe a relationship between sensory evaluation results and acidity, FI, and cut-test values. Remarkably, for the 6-hour PT samples fermented for 4 days, the differences in FQ and GQ scores compared to the 4-hour and 6-hour PT treatments fermented for 6 days were deemed minimal for practical application on-farm. Howbeit, the superior flavour attributes displayed by the predried samples could, among others, be attributed to the potentially higher loads of microbial inoculum on the pulp surface after prolonged bean exposure during predrying. Besides, yeasts have been reported to contribute to bean flavour quality by generating several fruity and floral volatile compounds through the Ehrlich pathway [62].

Analysis of variance (ANOVA) revealed significant (*p* < 0.05) differences in the GQ of beans with PT and FD, as well as their interactions (Table 7). Regression analysis indicated that the linear and quadratic effects of PT and FD significantly (*p* < 0.05) influenced the overall flavour quality of beans (Table 3). However, there were no significant (*p* < 0.05) interaction effects between PT and FD. The *R*^2^ value was 0.6882, indicating that the model could account for over 68% of the changes in the GQ of the beans.

### 3.4. Principal Component (PCA) and Correlation Analysis

**Figure 6:**
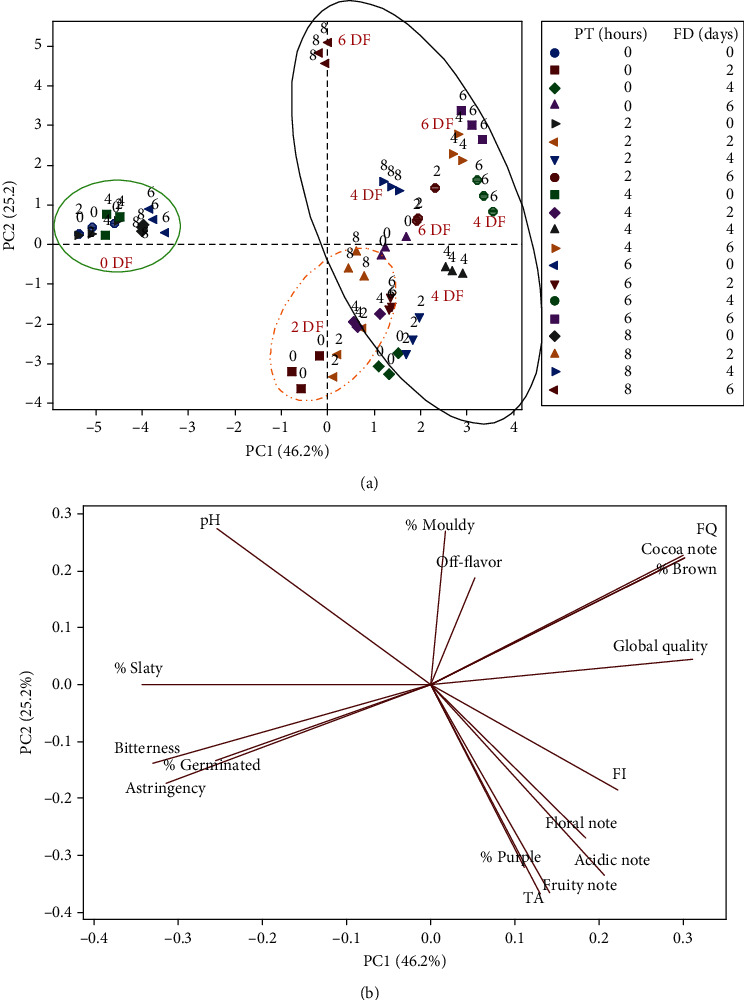
Principal component analysis (PCA). (a) Score plot from raw cocoa beans predried at 0, 2, 4, 6, and 8 hours and fermented for 0 (0DF), 2 (2DF), 4 (4DF), and 6 (6DF) days. (b) Loading plot for pH, titratable acidity (TA), cut-test score (% slaty beans, % purple beans, % brown bean, % germinated beans, % mouldy beans, and fermentation quality (% purity)), and flavour quality score (cocoa note, fruity note, floral note, acidity, astringency, bitterness, off-flavour, and overall flavour (global) quality (GQ)).

To visualize the distinct relationship between the various levels of PT treatments and the quality-indicating parameters assessed during cocoa bean fermentation, PCA was conducted. The distribution of scores and loading plot of the first two principal components (PC1 = 46.2% and PC2 = 25.2%) is illustrated in Figures 6(a) and 6(b), collectively explaining 71.5% of the total variability.

PC1 discriminated the predried cocoa beans during fermentation into 3 groups. Initially, at 0-day fermentation, the predried beans clustered closely together. Subsequently, after 2 days of fermentation, the 8-hour predried beans were more distinct from the 4 to 6-hour predried beans, which were also differentiated from the 0 to 2-hour predried beans. As fermentation progressed from the 4th to 6th days of fermentation, the predrying treatment levels were clearly differentiated.

The samples in the first group (negative axis) were characterized by high nib pH levels, incomplete fermentation, and a high occurrence of slaty and germinated beans. Liquors made from these beans exhibited extreme bitterness and astringency, with low cocoa notes and overall flavour quality. In the middle group, the 0-hour predried beans were associated with high levels of purple beans, nib TA, and fruity notes. Beans predried for 2–6 hours were predominantly characterized by high cocoa and other desirable flavour notes. Interestingly, and incongruent with earlier results, the 8-hour predried bean samples did not correlate with the assessed quality parameters. In the latter group (situated on the positive axis of PC1), the 0 and 2-hour predried beans fermented for 4 days exhibited low FI and high pH values, while those fermented for 6 days did not show a strong association with any measured quality parameters. Beans predried for 4 and 6 hours and fermented for 4 days correlated strongly with high overall flavour quality, while beans fermented for 6 days were, in addition, characterized by high % brown beans, % purity, and cocoa flavour notes. Conversely, beans predried for 8 hours and fermented for 4 and 6 days showed a strong correlation with off-flavour notes and a high occurrence of mouldy beans, respectively.

PC2, accounting for 25.2% of the total variance, discriminated between fermentation durations of 0 days, 2 days, and 4–6 days. Samples in the former group (0 days) were mainly characterized by high pH and a high occurrence of slaty and germinated beans. Liquors made from them were associated with an unpleasantly bitter and extremely astringent taste. The 2-day fermented beans were strongly characterized by a high TA content and a high occurrence of purple beans. Liquors made from them were highly associated with desirable flavour attributes such as floral and fruity notes, albeit with a high presence of acidic flavour notes. The sample in the latter group exhibited high fermentation quality due to the high abundance of brown beans, high FI values, and low occurrence of defective beans such as slaty, purple, germinated, and mouldy beans. Liquors made from the samples were strongly associated with high GQ due to the high cocoa notes, moderate floral and fruity attributes, and reduced acidity, bitterness, and astringent taste they exhibited. Conversely, samples predried for 8 hours and fermented for 6 days were characterized by high mouldy content and extreme off-flavour notes due to overrespiration and overfermentation [36].

The results indicate that predrying freshly harvested cocoa beans for 4–6 hours and fermenting for 4–6 days improves the fermentation quality by enhancing the fermentation effectiveness and wholesomeness of the beans. More so, the formation of flavour precursor compounds is stimulated, resulting in enhanced positive flavour attributes, and reduced undesirable flavour notes, ultimately enhancing the overall flavour (global) quality of the beans.

### 3.5. Optimization of Predrying and Fermentation Conditions

Table 8 shows the experimental data used in the model fitting. The optimized processing conditions were PT and FD of 6 hours and 6 days, respectively. These optimized processing conditions resulted in a composite desirability of 0.891.

## 4. Conclusion

Predrying and fermentation influenced the acidification, fermentation quality, and overall flavour quality of cocoa beans. Predrying improved the pH, acidity, fermentation effectiveness, bean wholesomeness, and global quality of the cocoa beans. Resultantly, predrying as a pulp preconditioning technique can be effectively employed as a viable alternative to the pod storage method conventionally used on-farm by small-scale farmers prior to cocoa bean fermentation. For optimal effects, however, a predrying duration of 6 hours followed by spontaneous heap fermentation for 6 days is recommended.

## Figures and Tables

**Table 1:** Effect of predrying on nib pH and titratable acidity (TA) during fermentation.

Predrying time (PT) (hours)	Fermentation duration (FD) (days)	pH	TA (meq NaOH/100 g)
0	0	6.61 ± 0.10^D^	0.023 ± 0.006^HIJ^
	2	4.95 ± 0.10^M^	0.173 ± 0.012^B^
	4	4.64 ± 0.10^N^	0.193 ± 0.006^A^
	6	5.28 ± 0.02^K^	0.083 ± 0.006^E^

2	0	6.66 ± 0.01^CD^	0.023 ± 0.05^HIJ^
	2	5.10 ± 0.01^K^	0.120 ± 0.010^D^
	4	4.97 ± 0.03^M^	0.150 ± 0.010^C^
	6	5.35 ± 0.02^J^	0.063 ± 0.015^F^

4	0	6.68 ± 0.01^BC^	0.030 ± 0.001^GHI^
	2	5.65 ± 0.01^IJ^	0.087 ± 0.006^E^
	4	5.36 ± 0.01^G^	0.110 ± 0.010^D^
	6	5.41 ± 0.02^I^	0.033 ± 0.006^GH^

6	0	6.71 ± 0.01^K^	0.027 ± 0.006^HI^
	2	5.76 ± 0.01^F^	0.053 ± 0.006^F^
	4	5.38 ± 0.02^IJ^	0.063 ± 0.006^F^
	6	5.48 ± 0.02^H^	0.020 ± 0.010^IJ^

8	0	6.72 ± 0.01^B^	0.033 ± 0.006^GH^
	2	6.17 ± 0.05^E^	0.040 ± 0.010^G^
	4	5.67 ± 0.03^G^	0.087 ± 0.006^E^
	6	7.33 ± 0.02^A^	0.013 ± 0.006^J^

Mean values having a common letter within the same column are not significantly different following a two-way ANOVA and LSD at the 5% level (*p* ≤ 0.05).

**Table 2:** ANOVA summary table showing *F*-ratios for variations in pH, titratable acidity (TA), fermentation quality (% purity), fermentation index (FI), and flavour (global) quality (GQ) of cocoa beans during fermentation.

Variables	pH	TA	% Purity	FI	GQ
Main effects					
A: PT (hours)	2661.40^∗^	202.80^∗^	259.89^∗^	147.62^∗^	142.44^∗^
B: FD (days)	7411.07^∗^	452.84^∗^	4854.65^∗^	440.25^∗^	169.29^∗^
Interaction					
A∗B	568.93^∗^	34.66^∗^	147.35^∗^	55.63^∗^	13.66^∗^
*R*^2^ (adjusted)	99.90%	97.75%	99.66%	97.75%	95.40%

^∗^Significant at *p* < 0.05.

**Table 3:** Regression coefficients and *R*^2^ values in the model for pH, titratable acidity (TA), fermentative quality (% purity), fermentation index (FI), and flavour (global) quality (GQ) of cocoa beans.

Variables	pH	TA	% Purity	FI	GQ
Constant	6.629^∗^	0.05531^∗^	-4.06^∗^	0.3988^∗^	23.98^∗^
*X*_1_	-0.0719^∗^	-0.01456^∗^	3..54^∗^	0.1303	2.821^∗^
*X*_2_	-0.9099^∗^	0.06397^∗^	15.24^∗^	0.3564^∗^	4.897^∗^
*X*_1_^2^	0.01417^∗^	0.001131^∗^	-0.454^∗^	-0.00889^∗^	-0.2567^∗^
*X*_2_^2^	0.10746^∗^	-0.009083^∗^	-0.845^∗^	-0.04244^∗^	-0.455^∗^
*X*_1_ × *X*_2_	0.02822^∗^	-0.001467^∗^	0.914^∗^	-0.01874^∗^	-0.1062
R^2^	0.8718	0.8353	0.9190	0.6158	0.6855

*X*_1_: predrying; *X*_2_: fermentation time. ^∗^Significant at *p* < 0.05.

**Table 4:** Effect of predrying on fermentation effectiveness (% brown beans), wholesomeness of beans, and fermentation quality (% purity) of cocoa beans during fermentation.

PT (hours)	FD (days)	Fermentation effectiveness			Bean wholesomeness			(% purity)
		Slaty (%)	Purple (%)	Brown (%)	Germinated (%)	Mouldy (%)	Weevily (%)	
0	0	87.7 ± 1.3^A^	0.0 ± 0.0^M^	0.0 ± 0.0^H^	12.3 ± 1.7^BCD^	0.0 ± 0.0^C^	0.0 ± 0.0	0.0 ± 0.0^J^
	2	26.7 ± 0.3^E^	28.0 ± 1.7^F^	31.3 ± 2.7^EF^	14.0 ± 4.7^BCD^	0.0 ± 0.0^C^	0.0 ± 0.0	31.3 ± 2.7^H^
	4	1.0 ± 0.3^GH^	46.0 ± 2.3^C^	42.3 ± 3.0^DE^	10.7 ± 4.7^CDE^	0.0 ± 0.0^C^	0.0 ± 0.0	42.3 ± 3.0^G^
	6	0.7 ± 0.7^H^	40.3 ± 2.3^CDE^	50.7 ± 0.7^D^	8.3 ± 2.3^DEF^	0.0 ± 0.0^C^	0.0 ± 0.0	50.7 ± 0.7^F^

2	0	83.0 ± 2.3^B^	1.7 ± 1.0^EF^	0.0 ± 0.0^H^	15.7 ± 3.0^ABC^	0.0 ± 0.0^C^	0.0 ± 0.0	0.0.7 ± 0.0^J^
	2	24.7 ± 2.7^E^	34.3 ± 2.0^E^	39.7 ± 1.0^DE^	1.3 ± 1.3^G^	0.0 ± 0.0^C^	0.0 ± 0.0	39.7 ± 1.0^G^
	4	0.0 ± 0.0^H^	52.7 ± 2.3^B^	43.0 ± 2.3^DE^	4.0 ± 1.0^EFG^	0.0 ± 0.0^C^	0.0 ± 0.0	43.0 ± 2.3^G^
	6	0.0 ± 0.0^H^	19.7 ± 2.7^GH^	75.6 ± 4.3^C^	5.0 ± 1.7^EFG^	0.0 ± 0.0^C^	0.0 ± 0.0	75.6 ± 4.3^D^

4	0	73.3 ± 3.7^C^	8.0 ± 1.3^KL^	0.0 ± 0.0^H^	18.7 ± 3.0^AB^	0.0 ± 0.0^C^	0.0 ± 0.0	0.0 ± 0.0^J^
	2	7.3 ± 3.0^F^	55.0 ± 2.0^B^	1.7 ± 1.3^H^	10.7 ± 3.0^CDE^	0.0 ± 0.0^C^	0.0 ± 0.0	1.7 ± 1.3^J^
	4	0.3 ± 0.3^H^	36.3 ± 3.5^DE^	63.0 ± 2.7^C^	0.7 ± 0.3^G^	0.0 ± 0.0^C^	0.0 ± 0.0	63.0 ± 2.7^E^
	6	0.0 ± 0.0^H^	0.0 ± 0.0^M^	98.0 ± 2.0^A^	2.0 ± 1.7^G^	0.0 ± 0.0^C^	0.0 ± 0.0	98.0 ± 2.0^AB^

6	0	70.7 ± 1.0^C^	27.3 ± 1.7^FG^	0.0 ± 0.0^H^	2.7 ± 1.0^G^	0.0 ± 0.0^C^	0.0 ± 0.0	0.0 ± 0.0^J^
	2	3.7 ± 1.0^FGH^	75.0 ± 4.0^A^	20.0 ± 2.0^FG^	1.3 ± 1.3^G^	0.0 ± 0.0^C^	0.0 ± 0.0	20.0 ± 2.0^I^
	4	0.0 ± 0.0^H^	4.7 ± 1.0^LM^	95.0 ± 1.0^AB^	1.0 ± 0.3^G^	0.0 ± 0.0^C^	0.0 ± 0.0	95.0 ± 1.0^B^
	6	0.0 ± 0.0^H^	0.3 ± 0.3^M^	99.7 ± 0.3^A^	0.0 ± 0.0^G^	0.0 ± 0.0^C^	0.0 ± 0.0	99.7 ± 0.3^A^

8	0	63.7 ± 1.7^D^	14.3 ± 0.7^IJ^	0.0 ± 0.0^H^	22.0 ± 1.3^A^	0.0 ± 0.0^C^	0.0 ± 0.0	0.0 ± 0.0^J^
	2	5.3 ± 0.7^FG^	41.7 ± 2.0^CD^	41.7 ± 2.3^DE^	11.7 ± 2.7^BCD^	0.0 ± 0.0^C^	0.0 ± 0.0	41.7 ± 2.3^G^
	4	1.0 ± 0.7^GH^	21.7 ± 1.3^GH^	75.3 ± 1.3^C^	2.0 ± 1.0^G^	1.3 ± 0.3^B^	0.0 ± 0.0	75.3 ± 1.3^D^
	6	1.0 ± 1.7^GH^	12.7 ± 2.3^JK^	82.7 ± 2.0^BC^	3.7 ± 1.3^FG^	2.0 ± 0.3^A^	0.0 ± 0.0	82.7 ± 2.0^C^

Values in the same column with the different superscript letters are statistically different. Values represent the mean values ± standard deviation of fermentation replicates.

**Table 5:** ANOVA summary table showing *F*-ratios for variations in fermentation effectiveness and bean wholesomeness during the fermentation of predried cocoa beans.

Variables	Fermentation effectiveness			Beans wholesomeness		% Purity
	Slaty	Purple	Brown	Germinated	Mouldy	
Main effects						
A: PT (hours)	160.15^∗^	15.68^∗^	265.66^∗^	28.23^∗^	240.25^∗^	259.89^∗^
B: FD (days)	9837.67^∗^	999.93^∗^	4977.49^∗^	62.07^∗^	93.58^∗^	4854.65^∗^
Interaction						
A∗B	56.63^∗^	258.89^∗^	151.07^∗^	7.47^∗^	93.58^∗^	147.35^∗^
*R*^2^ (adjusted)	99.81%	99.05%	99.67%	86.24%	97.55%	99.66%

^∗^Significant at *p* < 0.05.

**Table 6:** Effect of predrying on flavour attributes and global quality (GQ) of beans during fermentation.

PT (hours)	FD (days)	Positive flavour attributes			Negative flavour attributes				GQ
		Cocoa flavour	Fruity flavour	Floral flavour	Bitterness	Acidity	Astringency	Off-flavour	
0	0	2.0 ± 0.0^J^	1.0 ± 0.0^I^	1.0 ± 0.0^I^	2.6 ± 0.5^I^	9.0 ± 0.0^A^	2.3 ± 0.5^I^	9.0 ± 0.0^A^	26.9 ± 3.6^I^
	2	2.9 ± 0.4^HI^	4.4 ± 0.5^BC^	4.3 ± 0.5^H^	4.3 ± 0.5^H^	4.6 ± 0.5^J^	3.3 ± 0.5^H^	9.0 ± 0.0^A^	32.7 ± 2.0^FG^
	4	4.7 ± 0.5^EF^	3.1 ± 0.7^DE^	2.9 ± 0.4^DE^	5.3 ± 0.5^EFG^	2.4 ± 0.5^K^	5.4 ± 0.5^DE^	9.0 ± 0.0^A^	32.9 ± 2.2^FG^
	6	6.3 ± 0.5^D^	1.9 ± 0.4^FG^	1.7 ± 0.5^FGH^	6.3 ± 0.5^BCD^	6.3 ± 0.5^FG^	6.9 ± 0.4^B^	9.0 ± 0.0^A^	38.3 ± 2.7^CD^

2	0	2.1 ± 0.4^IJ^	1.0 ± 0.0^I^	1.0 ± 0.0^I^	2.7 ± 0.5^I^	9.0 ± 0.0^A^	2.4 ± 0.5^HI^	9.0 ± 0.0^A^	27.3 ± 3.5^I^
	2	3.9 ± 0.4^FG^	5.3 ± 0.5^A^	5.1 ± 0.4^AB^	4.7 ± 0.5^GH^	5.6 ± 0.5^HI^	4.4 ± 1.0^G^	9.0 ± 0.0^A^	37.9 ± 1.7^CD^
	4	5.3 ± 0.5^E^	3.9 ± 0.9^BC^	3.3 ± 0.5^D^	5.9 ± 0.7^CDE^	4.4 ± 0.5^J^	5.7 ± 0.5^DE^	9.0 ± 0.0^A^	37.4 ± 1.9^CD^
	6	6.7 ± 0.5^CD^	2.1 ± 0.7^FG^	1.9 ± 0.4^FGH^	6.7 ± 0.5^ABC^	6.6 ± 0.5^EFG^	7.1 ± 0.4^B^	9.0 ± 0.0^A^	40.1 ± 2.7^BC^

4	0	2.7 ± 0.5^IJ^	1.0 ± 0.0^I^	1.0 ± 0.0^I^	3.0 ± 0.0^I^	9.0 ± 0.0^A^	2.6 ± 0.5^HI^	9.0 ± 0.0^A^	28.3 ± 3.5^I^
	2	4.0 ± 0.0^FG^	4.7 ± 0.5^AB^	5.4 ± 0.5^A^	5.1 ± 0.4^FGH^	6.0 ± 0.6^GH^	4.6 ± 0.5^FG^	9.0 ± 0.0^A^	38.9 ± 1.7^BC^
	4	6.3 ± 0.5^D^	3.7 ± 0.5^CD^	3.6 ± 0.8^CD^	6.7 ± 0.5^AB^	5.0 ± 0.6^IJ^	6.0 ± 0.0^CD^	9.0 ± 0.0^A^	40.3 ± 1.9^AB^
	6	7.9 ± 0.7^AB^	2.3 ± 0.0^EF^	2.0 ± 0.0^FG^	7.0 ± 0.0^AB^	7.4 ± 0.5^CD^	7.4 ± 0.5^B^	9.0 ± 0.0^A^	43.0 ± 2.8^AB^

6	0	2.9 ± 0.4^HI^	1.3 ± 0.5^GHI^	1.3 ± 0.5^GHI^	3.0 ± 0.0^I^	9.0 ± 0.0^A^	2.7 ± 0.5^H1^	9.0 ± 0.0^A^	29.1 ± 3.4^HI^
	2	5.0 ± 0.6^E^	2.4 ± 0.5^D^	5.0 ± 0.5^A^	5.6 ± 0.5^CDE^	6.9 ± 0.4^DEF^	5.6 ± 0.0^DE^	9.0 ± 0.0^A^	41.0 ± 1.7^AB^
	4	7.4 ± 0.5^BC^	1.6 ± 0.5^EF^	4.3 ± 1.0^BC^	6.9 ± 0.4^AB^	6.0 ± 0.6^GH^	7.3 ± 0.8^B^	9.0 ± 0.0^A^	43.1 ± 2.2^AB^
	6	8.3 ± 0.5^A^	0.6 ± 0.0^GHI^	2.0 ± 0.5^EF^	7.3 ± 0.5^A^	7.9 ± 0.4^BC^	8.4 ± 0.5^A^	9.0 ± 0.0^A^	44.6 ± 3.1^A^

8	0	3.7 ± 0.5^GH^	1.6 ± 0.5^FGH^	1.3 ± 0.5^GHI^	3.4 ± 0.5^I^	9.0 ± 0.0^A^	3.0 ± 0.0^HI^	9.0 ± 0.0^A^	31.0 ± 3.3^GH^
	2	5.4 ± 0.5^E^	2.0 ± 0.0^FG^	5.4 ± 0.5^A^	5.7 ± 0.5^DEF^	7.3 ± 0.5^CDE^	5.3 ± 0.5^DE^	8.3 ± 0.5^B^	39.4 ± 2.0^BC^
	4	6.3 ± 0.5^D^	1.1 ± 0.4^HI^	3.4 ± 0.5^D^	6.0 ± 0.0^CDE^	8.3 ± 0.5^AB^	5.1 ± 0.3^EF^	5.9 ± 0.7^C^	36.1 ± 2.3^DE^
	6	6.9 ± 0.5^CD^	0.9 ± 0.4^I^	1.1 ± 0.4^HI^	6.6 ± 0.5^ABC^	8.4 ± 0.5^A^	6.6 ± 0.3^BC^	4.0 ± 0.6^D^	34.7 ± 3.0^EF^

Flavour attributes considered negative (i.e., acidity, astringency, bitterness, and off-flavour) were scored in quality inverse to their perceived taste scoring on a quality scale of 0 (bad) to 10 (outstanding). On a bitter note, a quality inverse score of 7.5 was considered optimal. A higher global quality score depicts superior final flavour quality. Values in the same column with different superscript letters are statistically different.

**Table 7:** ANOVA summary table showing *F*-ratios for variations in flavour score of sensory attributes during fermentation of predried cocoa beans.

Variables	Positive flavour attributes			Negative flavour attributes			Global quality
	Cocoa flavour	Fruity note	Floral note	Acidity	Bitter note	Astringent	
Main effects							
A: predrying time (hours)	27.70^∗^	23.39^∗^	5.42^∗^	67.59^∗^	8.86^∗^	14.19^∗^	11.12^∗^
B: fermentation duration (days)	235.04^∗^	95.58^∗^	205.98^∗^	210.31^∗^	150.9^∗^	219.45^∗^	57.77^∗^
Interaction							
A∗B	2.96^∗^	6.37^∗^	1.35	12.56^∗^	1.36	3.51^∗^	2.47^∗^
*R*^2^ (adjusted)	93.4%	88.1%	91.5%	94.6%	89.2%	92.6%	79.5%

^∗^Significant at *p* < 0.05.

**Table 8:** Design of experiment for process factors (*X*) and their responses (*Y*) for predrying time and fermentation duration.

Runs	Factor^∗^		Response (*Y*)^∗∗^			
	*X*_1_	*X*_2_	pH	FI	% purity	GQ
1	0	0	6.67	0.668	0.0	26.9
2	0	0	6.50	0.671	0.0	23.3
3	0	0	6.67	0.669	0.0	30.5
4	0	2	4.96	0.853	29.0	32.7
5	0	2	4.95	0.857	30.7	30.7
6	0	2	4.94	0.862	34.0	34.7
7	0	4	4.65	1.085	40.0	32.9
8	0	4	4.63	1.088	41.3	35.1
9	0	4	4.65	1.086	46.0	30.7
10	0	6	5.26	1.026	50.3	38.3
11	0	6	5.30	1.026	50.7	41.0
12	0	6	5.28	1.025	53.3	35.6
13	2	0	6.66	0.669	0.0	27.3
14	2	0	6.65	0.671	0.0	23.8
15	2	0	6.67	0.673	0.0	30.8
16	2	2	5.10	0.896	29.0	37.9
17	2	2	5.09	0.899	31.0	36.2
18	2	2	5.11	0.895	35.0	39.6
19	2	4	4.94	1.104	41.3	37.4
20	2	4	4.97	1.107	42.3	35.5
21	2	4	4.99	1.109	45.7	39.3
22	2	6	5.35	1.035	70.7	40.1
23	2	6	5.36	1.036	77.0	42.8
24	2	6	5.33	1.036	79.0	37.4
25	4	0	6.68	0.675	0.0	28.3
26	4	0	6.68	0.676	0.0	31.8
27	4	0	6.69	0.676	0.0	24.8
28	4	2	5.66	0.962	30.0	38.9
29	4	2	5.64	0.959	31.7	37.2
30	4	2	5.64	0.961	33.0	40.6
31	4	4	5.37	1.287	60.3	40.3
32	4	4	5.35	1.275	63.3	42.2
33	4	4	5.36	1.256	65.7	38.4
34	4	6	5.39	1.045	95.7	43.0
35	4	6	5.42	1.048	98.7	45.8
36	4	6	5.41	1.049	99.3	40.2
37	6	0	6.71	0.772	0.0	29.1
38	6	0	6.72	0.769	0.0	32.5
39	6	0	6.71	0.769	0.0	25.7
40	6	2	5.75	1.289	18.0	41.0
41	6	2	5.77	1.299	21.0	42.7
42	6	2	5.75	1.297	21.3	39.3
43	6	4	5.39	1.302	94.0	43.1
44	6	4	5.37	1.303	95.0	45.3
45	6	4	5.38	1.303	95.7	40.9
46	6	6	5.48	1.066	99.3	44.6
47	6	6	5.48	1.068	99.7	47.7
48	6	6	5.49	1.068	99.7	41.5
49	8	0	6.72	0.812	0.0	31.0
50	8	0	6.73	0.808	0.0	34.3
51	8	0	6.72	0.809	0.0	27.7
52	8	2	6.15	1.299	40.0	39.4
53	8	2	6.13	1.303	41.0	41.4
54	8	2	6.23	1.298	44.3	37.4
55	8	4	5.71	1.189	74.0	36.1
56	8	4	5.65	1.186	75.0	38.4
57	8	4	5.66	1.186	75.3	33.8
58	8	6	7.35	0.099	81.0	34.7
59	8	6	7.32	0.097	81.7	37.7
60	8	6	7.33	0.099	85.0	31.7

^∗^Factor: *X*_1_ (predrying time, hours) and *X*_2_ (fermentation duration, days). ^∗∗^Response (*Y*): pH; fermentation index, FI; fermentation quality, % purity; overall flavour (global) quality, GQ.

## Data Availability

The data used to support the findings of this study are included within the article. However, any other information required is available from the corresponding author upon request.
